# Deep learning approach for automatic out-of-plane needle localisation for semi-automatic ultrasound probe calibration

**DOI:** 10.1049/htl.2019.0075

**Published:** 2019-12-02

**Authors:** Leah A. Groves, Blake VanBerlo, Terry M. Peters, Elvis C.S. Chen

**Affiliations:** 1 School of Biomedical Engineering, University of Western Ontario, London, Ontario, Canada; 2 Robarts Research Institute, University of Western Ontario, London, Ontario, Canada; 3 Schulich School of Medicine, University of Western Ontario, London, Ontario, Canada; 4 Medical Biophysics, University of Western Ontario, London, Ontario, Canada

**Keywords:** medical image processing, calibration, learning (artificial intelligence), needles, image registration, mean square error methods, biomedical ultrasonics, convolutional neural nets, out-of-plane needle localisation, semiautomatic ultrasound probe calibration, deep learning algorithm, needle reflection, probe calibration algorithm, convolutional neural network, automatic centroid localisation algorithm, probe calibrations, pixel localisation, semiautomatic implementation, automatic needle centroid localisation, target registration errors, calibration method, size 6.0 cm, size 4.0 cm to 8.0 cm

## Abstract

The authors present a deep learning algorithm for the automatic centroid localisation of out-of-plane US needle reflections to produce a semi-automatic ultrasound (US) probe calibration algorithm. A convolutional neural network was trained on a dataset of 3825 images at a 6 cm imaging depth to predict the position of the centroid of a needle reflection. Applying the automatic centroid localisation algorithm to a test set of 614 annotated images produced a root mean squared error of 0.62 and 0.74 mm (6.08 and 7.62 pixels) in the axial and lateral directions, respectively. The mean absolute errors associated with the test set were 0.50 ± 0.40 mm and 0.51 ± 0.54 mm (4.9 ± 3.96 pixels and 5.24 ± 5.52 pixels) for the axial and lateral directions, respectively. The trained model was able to produce visually validated US probe calibrations at imaging depths on the range of 4–8 cm, despite being solely trained at 6 cm. This work has automated the pixel localisation required for the guided-US calibration algorithm producing a semi-automatic implementation available open-source through 3D Slicer. The automatic needle centroid localisation improves the usability of the algorithm and has the potential to decrease the fiducial localisation and target registration errors associated with the guided-US calibration method.

## 1 Introduction

**Fig. 1:**
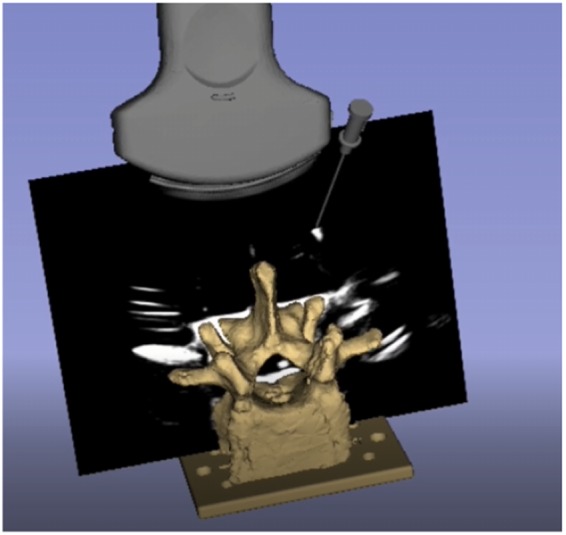
US image registered to the tracker's coordinate system. Accuracy is indicated through the alignment of the virtual models and their reflections on the US image

Ultrasound (US) scanners are common in image-guided interventions as they produce real-time imaging without exposing the patient to harmful ionising radiation [1]. Mixed-reality US-guided surgical navigation systems aim to improve the usability of US-guided interventions by using a 3D virtual environment to provide a visual relationship between tracked surgical instruments and real-time US images [2]. These systems rely on US probe calibration to establish the spatial transformation between the US image and a tracking sensor attached to the transducer [3]. Using the sensor fixed to the transducer as a reference sensor, the calibration can be used to provide the relationship between other tracked tools and the US image [3], as depicted in Fig. 1.

US probe calibration remains an active research area in spite of the development of multiple US probe calibration methods. Two of the main barriers to the translation of US probe calibration methods from the labs to the clinic are the requirement of specific calibration phantoms and difficult calibration techniques relying on knowledge of US physics [4]. The US probe calibration method that is used for this work is based on the Guided US Calibration (GUSCAL), which formulates the US calibration as a Procrustean point-to-line registration problem [5]. It is quick and effective, enabling even novices to complete successful calibrations in 3–5 min [6]. The original formulation of this approach used a tracked and calibrated straw phantom imaged in water [5]. However, in recent work to improve the usability and accessibility of this algorithm, the calibrated straw phantom was replaced with a tracked needle [6]. Magnetically tracked surgical needles are common with many tracking systems, can be purchased pre-calibrated from the manufacturers, and are readily used in the field of computer-assisted surgical navigation. The tracked needle GUSCAL method requires localisation of the centroid of out-of-plane needle reflections, where, rather than the entire needle being in the US plane, it is inserted at an oblique angle intersecting the US plane, producing a cross-sectional reflection of the needle shaft on a black background [5].

The major limitation with the GUSCAL approach is the high fiducial localisation error (FLE) produced as a result of users incorrectly localising the needle centroid [6]. Due to the blur of the US image in the far-field, the needle's reflection is significantly amplified within the lower portion of the US image relative to the reflections produced in the upper portion [6], particularly for lower quality US machines. The inconsistent appearance of needle reflections throughout the image negatively affects the accuracy of the centroid localisation, with novice users experiencing difficulties correctly determining the pixel that best represents the centroid of the needle reflection [6]. The FLE propagates into target registration error (TRE), which adversely affects the accuracy of the registration between the US image and the spatial tracking system [6]. Manual localisation is a barrier to use for novice users and negatively affects the accuracy of all users’ calibrations. An automatic localisation algorithm has the potential to improve the FLE and thus the accuracy and usability of the GUSCAL method.

**Fig. 2:**
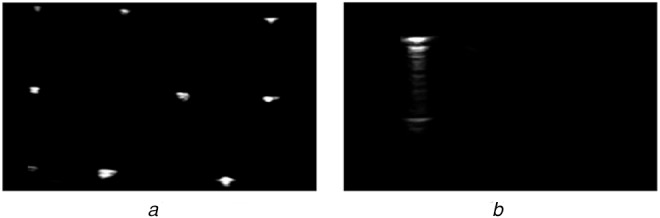
*a* Appearance of the US image required for the *Z*-phantom calibration approach *b* Appearance of the US image required for the GUSCAL approach

Due to the unique appearance and lack of tissue present in the images required for the augmented GUSCAL approach, there has been no work to automate out-of-plane needle centroids on a uniform black background. However, one common calibration algorithm requires the imaging of a *Z*-phantom comprising of thin wires in the form of a ‘*Z*’ [7]. Imaging these wires submerged in water produces three bright co-linear point reflections on a black background, as depicted in Fig. 2*a*, which resembles the images required for the GUSCAL approach as depicted in Fig. 2*b*. While an automatic approach to segmenting the reflections of the thin wires has been implemented [8], this approach relies on the standard and known geometry of a *Z*-phantom, as the image always contains three co-linear reflections [8]. Furthermore, the GUSCAL approach involves inserting the needle at an oblique angle such that it fully intersects the US image plane, producing a more amplified reflection compared to the thin wires. The difference in appearance in the images and methods required for the GUSCAL approach compared to the *Z*-phantom method has motivated the development of the solution described below.

Deep learning solutions have been successful for many image segmentation and data regression problems due to their efficient formulas, particularly in medical image analysis [9, 10]. This motivates us to apply and evaluate these techniques for the problem of localising out-of-plane US needle reflections with a uniform black background. This Letter presents a deep convolutional neural network (CNN) model that performs automatic needle localisation. The main contribution of this work is the development and validation of a CNN model to automatically localise out-of-plane needle reflections for a US calibration method with anisotropic scaling. This network has been integrated into an open-source semi-automatic US probe calibration that offers improved accuracy and usability compared to its manual localisation counterpart. An open-source module that incorporates both the manual and automatic methods for US probe calibration is available in 3D Slicer, which is available for download at https://slicer.org. This work forms the basis of high accuracy and fully automatic US probe calibration method.

## 2 Materials and methods

### 2.1 Data collection

An Ultrasonix Sonix Touch (BK Medical, USA) US scanner with an L14-5 linear probe was used for this work. The magnetic tracking system used for data collection was the NDI Aurora Tabletop tracking system (NDI, Waterloo, Canada). Prior to data collection, the US probe used for the experiment was carefully calibrated by an expert to the Aurora's coordinate system through the GUSCAL approach with manual localisation. The calibration is computed between corresponding pairs of points and lines. The points are generated by inserting a tracked needle out-of-plane, such that the reflection represents the cross-section of the needle shaft. The centroid of the needle reflection is localised manually by the user to produce a single pixel point [5]. The line is formed by the position of the needle tip and the direction vector associated with the orientation of the needle at the time the image was captured [5]. The calibration is solved using an iterative solution, where the TRE produced after each point and line collection plateaus to a stable minimum after ∼12 measurements [5].

**Fig. 3:**
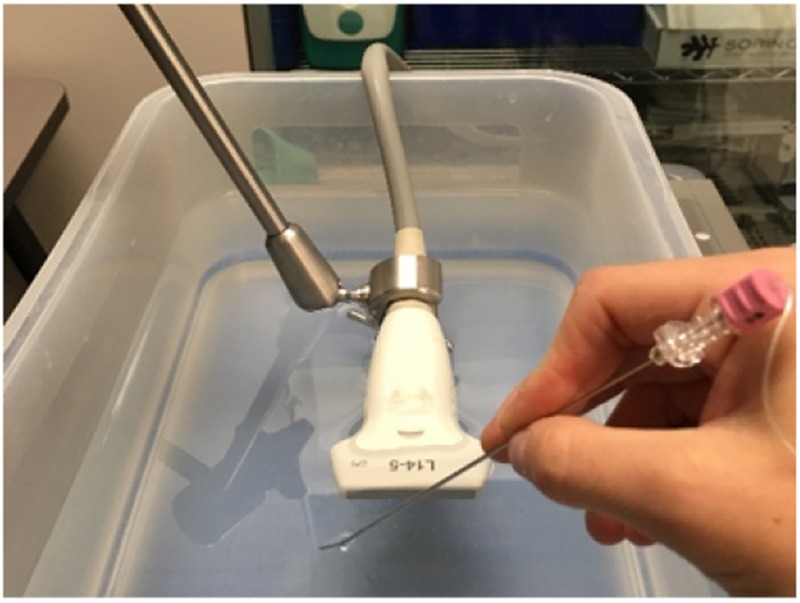
Data collection set-up depicting the fixed calibrated US probe (tracking sensor is embedded within the probe) and the needle intersecting the US beam at an oblique angle

The calibrated L14-5 US probe acquires images using the PLUS Server [11]. As the calibrated US images are registered to the spatial tracking system, the relationship between the tracked needle and the US image is known. During data collection, the transducer face of the US probe is submerged in water and the needle imaged throughout the entire 6 cm image depth with various orientations, as depicted in Fig. 3. A training set of 3825 images, a validation set of 1519 images, and a test set of 614 images were collected over three independent sessions. These datasets were culled by an expert to remove images where the centroid position did not align with the needle reflection or where the needle was outside of the bounds of the image. The culled, annotated datasets were used for training and evaluation of the deep learning localisation model.

### 2.2 Dataset preprocessing

The raw US images within the training, validation, and test sets were acquired as 8-bit 356 × 589 pixel images. These images were resized to 128 × 128 pixels with bilinear interpolation to reduce variability and decrease the number of trained parameters in the neural network. The pixel values were normalised to the range of [0, 1]. To generate ground-truth annotations, the inverse of the calibration matrix was applied to the matrix representing the needle's pose producing a 2D pixel location representing the centroid of the needle reflection. Thus, using the known calibration the intersection between the tracked needle and the US images was computed, serving as the ground-truth for training, validation, and test sets. This process generated a label defining the coordinates of the centroid of the needle's reflection for each image in the set, producing corresponding sets of images and single-pixel labels. Each label is an (*x*, *y*) coordinate pair. The *x*-value was scaled from the range of integers in [0, 355] to the continuous range of [−1, 1]. The *y*-value was scaled from the range of integers in [0, 588] to the continuous range of [−1, 1]. The output of the algorithm returns pixel locations in the range [−1, 1]. A scaling value was then applied to these output locations and then rounded to produce new integer pixel coordinates that corresponded to the centroid of the reflection in the original sized image.

### 2.3 Neural network architecture

**Fig. 4:**
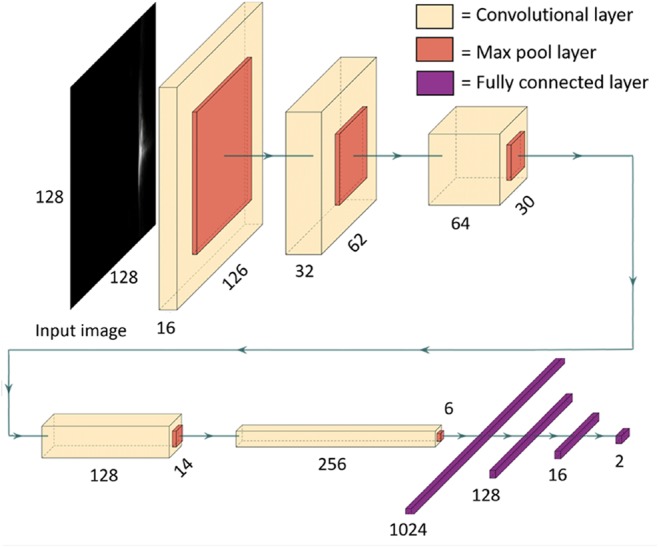
Diagram depicting the architecture of the neural network model used to predict the coordinates of the centroid of a US needle reflection

Automatically localising the centroid of the needle in a US image can be thought of as a keypoint localisation problem. A facial keypoint detection algorithm [12] motivated the architecture presented in this Letter. CNN was designed to receive a US image as its input and predict coordinates over a continuous range. An iterative training and testing process was implemented to assess which architecture worked best for the required task. The final network consists of five alternating convolutional and max-pooling layers, followed by four fully connected layers. The output layer is a fully-connected layer with two units, which outputs the regressed coordinates of the centroid of the US needle. The depth of the convolutional layers increases with each successive layer. The number of filters in the first layer is 16, and this number doubles with each successive convolutional layer. The first convolutional layer uses filters of size 3 × 3, and all successive convolutional layers use filters of size 2 × 2. All convolutional layers use a stride of 1. The initial parameters of the convolutional layer were determined using Glorot uniform initialisation [13]. All max-pooling layers use a pool size of 2 × 2 and a stride of 1. The activation function used in all pre-output layers is the Leaky Rectified Linear Unit (Leaky ReLU) function, which was chosen over Rectified Linear Unit (ReLU) because it converges faster during training [14]. The output layer has no activation function, i.e. it is an unmodified linear output. The output layer predicts the coordinates of the centroid of the needle in the image, whose values are bounded by [−1, 1]. The dataset used to train this network did not contain images in which the centroid was outside of the image. Since the output layer is linear, the network may predict a coordinate pair outside of this range. However, should this scenario occur, the 3D Slicer implementation only accepts predicted centroid coordinates within the original image bounds and would return an error, prompting the user to input a new image. In total, the model contains 2 668 338 trained parameters. A diagram of the model's specific architecture is depicted in Fig. 4.

### 2.4 Neural network training

**Fig. 5:**
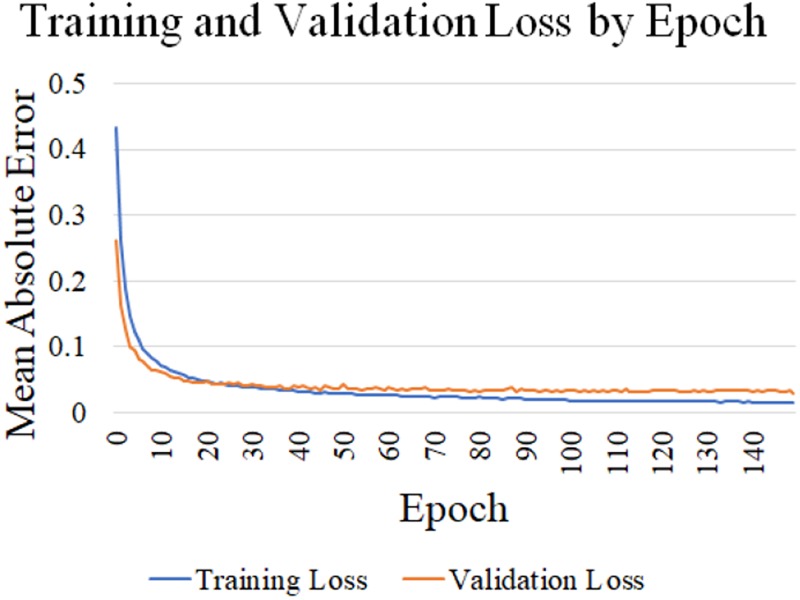
Graph displaying the change in training and validation loss (MAE) throughout the training process. The loss is calculated in terms of the output of the neural network, which is an (x, y) coordinate pair with values in [−1, 1]

The batch size was 128 and the model was trained for 150 epochs to minimise the mean absolute error (MAE) loss function. We employed the Adam optimisation algorithm [15], with $\alpha = 1 \times 10^{ - 4}$ for epochs 1–100, and $\alpha = 7 \times 10^{ - 5}$ for epochs 101–150. To reduce overfitting, L2 regularisation with $\lambda = 1 \times 10^{ - 5}$ was applied in all convolutional layers. These hyperparameters were determined iteratively in a heuristic manner. One hyperparameter was varied at a time while all others were held constant. As the training, validation and test sets were recorded during independent sessions, they were not a random split from the complete dataset. Fig. 5 demonstrates the minimisation of the loss function over the training process.

Training experiments were completed using hardware, including an Intel i7-7800X CPU at 3.5 GHz and an Nvidia Titan Xp GPU with 12 GB of memory. The code was written in Python and the model architecture was defined using the Keras API with the TensorFlow backend.

## 3 Results

### 3.1 Centroid localisation accuracy

The accuracy of the needle centroid localisation was evaluated using a test set, obtained independently from both the training and validation sets. For each image the root mean squared error (RMSE) and MAE between the previously labelled and automatically generated pixel locations was calculated. Both similarity metrics provide distances between the labelled and automatically generated pixel coordinates obtained from the 8-bit 356 × 589 pixel images. To convert from pixel values to millimetres, the scaling factors obtained from a calibration are required. A major issue in US probe calibration research is the inability to produce a ground-truth calibration. If a method existed to generate a calibration with gold-standard certainty, the relationship between the tracking system and the US image would be known and there would be no need for US calibration research. Therefore, the scaling values representing the pixel spacing were obtained by performing five careful manual calibrations for a single US probe at 6 cm. The scaling factors in the axial and lateral directions were extracted from each transformation matrix using single value decomposition on the 3 × 3 anisotropic scaling and rotation matrix.

**Fig. 6:**
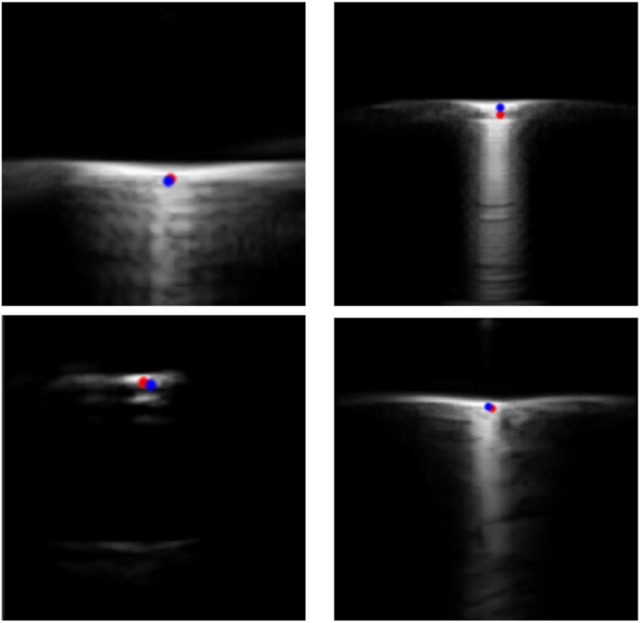
Four example images extracted from the test set that are representative of images that would be obtained within the GUSCAL calibration approach. The manual ground-truth localisations are indicated in red and the automatic localisations are indicated in blue, providing a visual representation of localisation accuracy

Four sample images extracted from the test set and the manual and automatic localisation are depicted in Fig. 6. The RMSE distance between the automatic and ground-truth segmented locations is presented in Table 1. The average RMSE for the test set was 0.62 and 0.74 mm (6.08 and 7.62 pixels) for the axial and lateral directions, respectively. The MAE and standard deviation of the test set was 0.50 ± 0.40 mm and 0.51 ± 0.54 mm (4.9 ± 3.96 and 5.24 ± 5.52 pixels) for the axial and lateral directions, respectively. As this solution localises a single-pixel coordinate, the most descriptive error metrics are physical and pixel-based distances between the labelled and automatic centroid positions. Localising the single-pixel needle centroid with absolute certainty is a difficult task, as user bias results in variability between expert users’ manual localisations.

**Table 1:** RMSE and the MAE between the ground-truth and automatically generated localisations from the test set summarised for the axial and lateral directions in pixels and millimetres

Metric	Axial (*X*)	Lateral (*Y*)
RMSE, pixels	6.08	7.62
RMSE, mm	0.62	0.74
MAE, pixels	4.9 ± 3.96	5.24 ± 5.52
MAE, mm	0.50 ± 0.40	0.51 ± 0.54

**Fig. 7:**
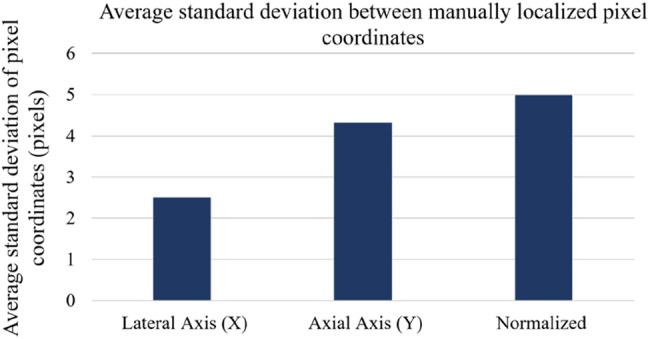
Average standard deviation of five expert users’ manual pixel localisations from ten images of needle reflections taken from the test set. This image set contained a dispersion of needle reflection positions throughout the lateral axis of the image

To analyse this variability, five users who were familiar with the calibration process manually selected the needle centroid on ten unique US images that were taken from the test set. The average standard deviation of the pixel's axial, lateral, and normalised locations was calculated and reported in Fig. 7. The variabilities in the lateral and axial directions were 2.5 and 4.3 pixels, respectively. This highlights the variability in centroid localisation, which could have an impact on the ground-truth labels used to produce the error metrics. The reported error is <1 mm for both error metrics and the network has produced visually acceptable calibrations. In future work, a comprehensive accuracy analysis of the semi-automatic calibration approach will be conducted. Alternatively, annotated datasets may be generated using simulated US physics to maximise the accuracy of the ground-truth data labels, thereby improving the accuracy analysis of the model.

### 3.2 Calibration accuracy

The accuracy of the pixel localisations is meaningful to describe the accuracy of the deep learning algorithm. However, the accuracy of the semi-automatic calibration is a more meaningful result. The semi-automatic implementation of the GUSCAL method is publically available in 3D Slicer. This program requires the user to stream the US images and the needle and probe tracking data into 3D Slicer, in real-time or through a recording. When the user has an image they deem appropriate for the calibration, a single button press or keyboard input is required by the user, which freezes the image and tracking streams, performs the automatic localisation of the needle reflection, and then resumes tracking streams. This process is completed almost instantaneously. The user repeats this process for 12–15 images, where the majority of images are collected with the needle intersecting the image plane at an oblique angle near the corners of the image. The output of this process is the calibration transformation matrix, which can be applied to the image to register it to the tracker's coordinate system.

**Fig. 8:**
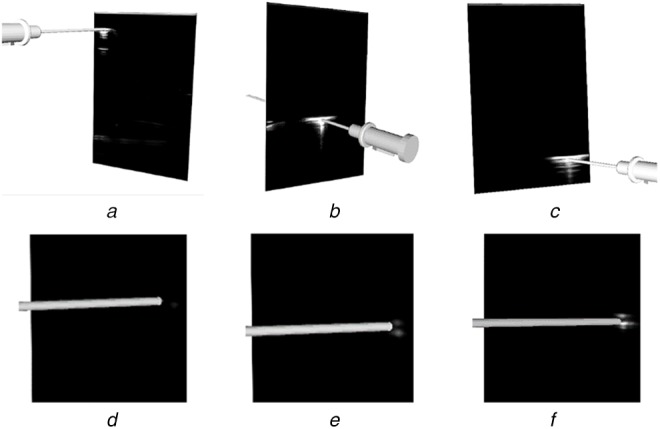
Output from the semi-automatic US probe calibration algorithm implemented in 3D Slicer at an imaging depth of 6 cm The figure depicts the accuracy in the axial and lateral directions through the alignment of the virtual needle with the centre of the reflection throughout the image, as depicted in *(a)*–*(c)* and Images *(d)*–*(f)* depict the accuracy in the elevation (*Z*) direction as the needle enters the image plane

**Fig. 9:**
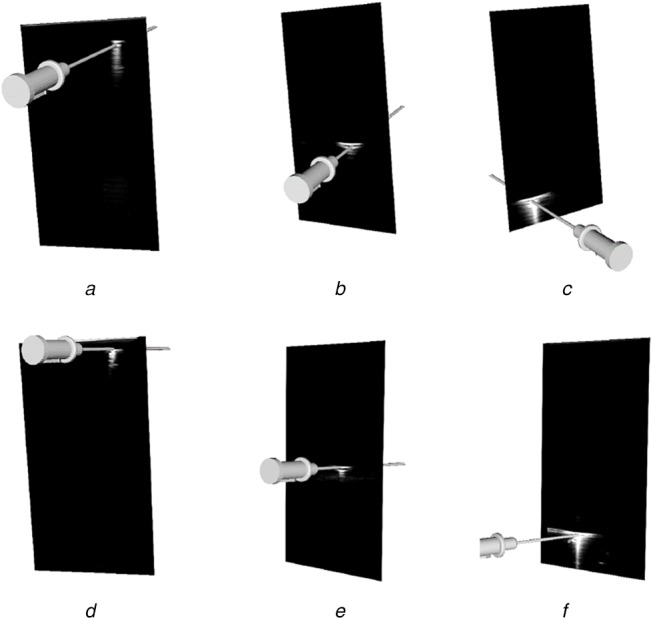
Output from the semi-automatic US probe calibration algorithm implemented in 3D Slicer at various imaging depths Images *(a)*–*(c)* have at an imaging depth of 7 cm. Images *(d)*–*(f)* have an imaging depth of 8 cm. All images depict the accuracy in the axial and lateral directions by the alignment of the virtual needle with the centre of the reflection at different lateral positions throughout the image

**Fig. 10:**
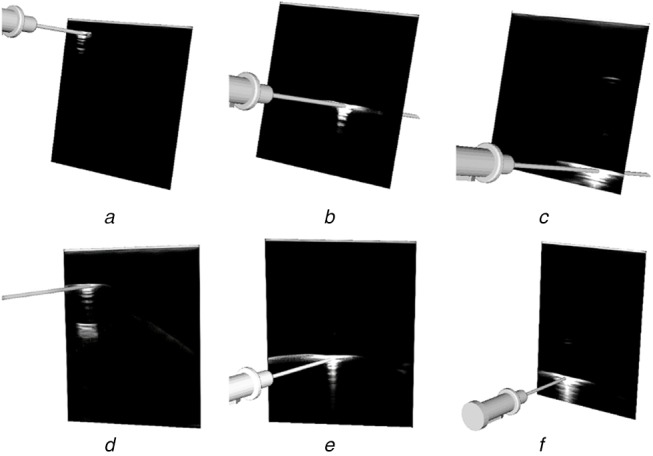
Output from the semi-automatic US probe calibration algorithm implemented in 3D slicer at various imaging depths Images *(a)*–*(c)* have an imaging depth of 4 cm. All images depict the accuracy in the axial and lateral directions by the alignment of the virtual needle with the centre of the reflection at different lateral positions throughout the image

Evaluating the accuracy of a US calibration is difficult as there is no means to generate ground-truth data. A visual representation of the accuracy was produced by showing the alignment of virtual models of tracked tools with the reflections produced by the real tool within the US image. Five calibration datasets were collected by recording the images of needle reflections and the respective needle pose with respect to the reference sensor fixed to the probe at imaging depths of 4–8 cm. These datasets were used to obtain calibrations using the semi-automatic GUSCAL calibration approach. A range of imaging depths was collected to show the ability of the deep learning model to generalise to depths other than the single imaging depth (6 cm) used to train the network. Visual depictions of the calibration accuracy, shown through the alignment between the virtual needle and the reflection within the image, for all the aforementioned imaging depths are presented in Figs. 8–10.

## 4 Discussion

Our approach to solving the problem of out-of-plane US needle centroid localisation for US probe calibration is accurate and reliable for a single US probe across a variety of imaging depths. Since US images captured at different depths have varying dimensions, the resize step of the image preprocessing enabled the network to accept images of different depths. Despite training the network for images captured at a depth of 6 cm, the network is capable of performing centroid localizations and therefore, calibrations at imaging depths ranging from 4–8 cm, as depicted in Figs. 8–10. Using a training set consisting only of images captured at a depth of 6 cm was sufficient for the network to generalise to other imaging depths at test time. This has provided motivation to augment the training set to include images captured at a range of depths to improve the accuracy of the model.

One limitation of this work is the inability to apply this trained network to other US probes, such as linear probes developed by other manufacturers, or curvilinear probes developed by the same manufacturer as the probe used to train the network. Future work could include reusing the neural network architecture (as depicted in Fig. 4) to train on annotated datasets comprising images from different probes and assessing the results. Large automatically annotated datasets can be produced using a calibrated US probe and the corresponding needle tracking data, with the process outlined in the methods. This allows for a simple workflow to develop deep learning models for an array of US probes. A large data set comprising annotated images from a range of US probe geometries and imaging depths could be compiled and used to re-train the network, such that it can be generalised for a range of US systems.

As the calibration phantom being used is a pre-calibrated needle, the only error introduced by the calibration phantom is the tracking error of the system, which has been reported by the manufacturer to be 1.2 mm for position and 0.5° for orientation for the specific needle phantom being used (https://www.ndigital.com/medical/products/aurora). Using a pre-calibrated needle produces a more usable system as no previous calibration steps are required prior to the probe calibration. Automating the needle reflection localisation intends to further improve the usability of this algorithm, as users only have to focus on collecting needle reflections and are not required to identify the centre of the needle reflection. Furthermore, in previous work, we found that the manual localisations result in FLE that propagates into TRE. Automating this process aims to improve the TRE and usability of the GUSCAL method. In future work, we aim to evaluate how usability was affected by automating the needle reflection.

Additional future work will focus on the development of a completely automatic calibration algorithm. We aim to develop a method in which the user can simply insert the tracked needle into the US beam, collecting a variety of needle reflections throughout the image. The automated calibration algorithm will be used to segment these reflections and use an outlier rejection approach to produce the best calibration possible from the recorded set of images. We believe this method would produce a highly usable and accurate algorithm, allowing for easy translation and use by novices. Other future work includes comprehensive accuracy validation for the GUSCAL method for both manual and automatic localisation approaches. Some potential future applications based on this semi-automatic US probe calibration method are US reconstruction for 3D modelling and surgical planning or US-guided needle insertions.

## 5 Conclusion

This work developed and assessed a deep learning framework for automatic centroid localisation of out-of-plane US needle reflections for the GUSCAL US probe calibration method. This method has been made open-source and can be used in the nightly release of 3D Slicer through the SlicerVASST extension (https://github.com/VASST/SlicerVASST). Please refer to the GitHub repository (https://github.com/VASST/AECAI.CNN-US-Needle-Segmentation) for the code required to replicate these results. The automatic localisations predicted by the deep learning model produce accurate calibrations for imaging depths ranging from 4–8 cm, despite having trained solely on images acquired at 6 cm. This method was able to produce localisation with an RMSE of 0.62 mm (6.4 pixels) and 0.74 mm (7.62 pixels) from the expert labelled locations in the axial and lateral directions, respectively. The MAE and standard deviations calculated between the ground-truth and automatic labelled were 0.5 ± 0.4 mm and 0.51 ± 0.54 mm (4.9 ± 3.96 pixels and 5.24 ± 5.52 pixels) in the axial and lateral directions, respectively. Overall, the accuracy of the automatic localisations of out-of-plane needle reflections is sufficient to provide visually accurate calibrations over a range of imaging depths, as depicted in Figs. 8–10. Future work includes retraining the model for other US probe geometries and developing a fully automated US calibration method. Thus, we provide a method to automatically localise the needle centroid with high accuracy. This work has the potential to improve the overall accuracy and usability of the GUSCAL US probe calibration method.

## References

[1] Noble J.A. Navab N. Becher H.: ‘Ultrasonic image analysis and image-guided interventions’, Interface. Focus., 2011, 1, (4), pp. 673–685 (doi: 10.1098/rsfs.2011.0025) 22866237 10.1098/rsfs.2011.0025 PMC3262276

[2] Peters T.M. Linte C.A. Yaniv Z.: ‘Mixed and augmented reality in medicine’ (CRC Press, Boca Raton, FL, USA, 2018)

[3] Hsu P.W. Prager R.W. Gee A.H.: ‘Freehand 3D ultrasound calibration: a review’, in ‘Advanced imaging in biology and medicine’ (Springer, Berlin, Heidelberg, 2009), pp. 47–84

[4] Mercier L. Langø T. Lindseth F.: ‘A review of calibration techniques for freehand 3-d ultrasound systems’, Ultrasound Med. Biol., 2005, 31, (2), pp. 143–165 (doi: 10.1016/j.ultrasmedbio.2004.11.001) 15708453 10.1016/j.ultrasmedbio.2004.11.001

[5] Chen E.C.S. Peters T.M. Ma B.: ‘Guided ultrasound calibration: where, how, and how many calibration fiducials’, Int. J. Comput. Assist. Radiol. Surg., 2016, 11, (6), pp. 889–898 (doi: 10.1007/s11548-016-1390-7) 27038966 10.1007/s11548-016-1390-7

[6] Groves L. Rankin A. Peters T.M.: ‘The effect of imaging and tracking parameters on ultrasound probe calibration robustness’, in Fei B. Linte C.A. (Eds.): ‘SPIE medical imaging 2019: image-guided procedures, robotic interventions, and modeling’, vol. 10951 (SPIE, Washington, DC, USA, 2019), p. 32

[7] Comeau R.M. Fenster A. Peters T.M.: ‘Integrated MR and ultrasound imaging for improved image guidance in neurosurgery’, in Hanson K.M. (Ed.): ‘SPIE medical imaging 1998: image processing’, vol. 3338 (SPIE, Washington, DC, USA, 1998), pp. 747–754

[8] Bartha L. Lasso A. Chen T.K.: ‘Automatic fiducial localization in ultrasound images for a thermal ablation validation platform’, in Wong K.H. Holmes III D.R. (Eds.): ‘SPIE medical imaging 2011: visualization, image-guided procedures, and modeling’, vol. 7964 (SPIE, Washington, DC, USA, 2011), p. 796421

[9] Liu S. Wang Y. Yang X.: ‘Deep learning in medical ultrasound analysis: a review’, Engineering, 2019, 5, pp. 261–275 (doi: 10.1016/j.eng.2018.11.020)

[10] Lee J.G. Jun S. Cho Y.W.: ‘Deep learning in medical imaging: general overview’, Korean J. Radiol., 2017, 18, (4), pp. 570–584 (doi: 10.3348/kjr.2017.18.4.570) 28670152 10.3348/kjr.2017.18.4.570 PMC5447633

[11] Ungi T. Lasso A. Fichtinger G.: ‘Open-source platforms for navigated image-guided interventions’, Med. Image Anal., 2016, 33, pp. 181–186 (doi: 10.1016/j.media.2016.06.011) 27344106 10.1016/j.media.2016.06.011

[12] Longpre S. Sohmshetty A.: ‘Facial keypoint detection’, Stanford University, Stanford, CA, USA, 2016

[13] Glorot X. Bengio Y.: ‘Understanding the difficulty of training deep feedforward neural networks’, Proc. of the 13th Int. Conf. on Artificial Intelligence and Statistics, Sardinia, Italy, 2010, vol. 9, pp. 249–256

[14] Maas A.L. Hannun A.Y. Ng A.Y.: ‘Rectifier nonlinearities improve neural network acoustic models’, 2013

[15] Kingma D.P. Ba J.: ‘Adam: A method for stochastic optimization’, Proc. of the 3rd Int. Conf. for Learning Representations (ICLR), San Diego, CA, USA, 2014, pp.1–15.

